# A model of processive walking and slipping of kinesin-8 molecular motors

**DOI:** 10.1038/s41598-021-87532-0

**Published:** 2021-04-13

**Authors:** Ping Xie

**Affiliations:** Key Laboratory of Soft Matter Physics, Institute of Physics, Chinese Academy of Sciences, Beijing, 100190 China

**Keywords:** Biophysics, Molecular biology

## Abstract

Kinesin-8 molecular motor can move with superprocessivity on microtubules towards the plus end by hydrolyzing ATP molecules, depolymerizing microtubules. The available single molecule data for yeast kinesin-8 (Kip3) motor showed that its superprocessive movement is frequently interrupted by brief stick–slip motion. Here, a model is presented for the chemomechanical coupling of the kinesin-8 motor. On the basis of the model, the dynamics of Kip3 motor is studied analytically. The analytical results reproduce quantitatively the available single molecule data on velocity without including the slip and that with including the slip versus external load at saturating ATP as well as slipping velocity versus external load at saturating ADP and no ATP. Predicted results on load dependence of stepping ratio at saturating ATP and load dependence of velocity at non-saturating ATP are provided. Similarities and differences between dynamics of kinesin-8 and that of kinesin-1 are discussed.

## Introduction

The superfamily of kinesin proteins consist of 14 families—kinesin-1 through kinesin-14—and ungrouped family termed as orphan kinesin^1^. They are molecular motors that make use of the free energy change arising from ATP hydrolysis to move on microtubule (MT), performing diverse cellular functions such as intracellular transport and regulation of MT dynamics^2,3^. Like kinesin-1 motors with their motor domains (or heads) in the N-terminus (called N-type kinesins), which can transport cargo by moving processively towards the plus end of MT (with a run length of the order of 1 µm)^4–6^, the N-type kinesin-8 motors such as Kip3 (budding yeast kinesin-8) and Kif18A (human kinesin-8) can also make processive plus-end-directed motion (with a run length of the order of 10 µm), depolymerizing MTs after they reach the plus end^7–12^.

For kinesin-1, it was determined that the dimeric motor can exert a force of about 6–8 pN and move in a hand-over-hand manner: during a forward step along a MT filament, the leading head binds fixedly to a MT-binding site, while the trailing head detaches from the previous rear MT-binding site, moves forward and then binds to the next front MT-binding site, becoming the new leading head^13–18^. For kinesin-8 such as Kip3 and Kif18A, it was shown that the dimers can only exert a very low force (with a stall force of only about 1 pN) and its superprocessive movement is frequently interrupted by brief stick–slip motion^19^. In the presence of saturating ADP and no ATP, Kip3 dimer shows rapid processive slip^20^.

From the theoretical point of view, many researchers focused on the modeling of kinesin-1 stepping^21–31^. However, few works have focused on kinesin-8. Although the slipping dynamics of kinesin-8 in the presence of saturating ADP and no ATP was studied theoretically^20^, the dynamics of slipping during the processive motion of the motor or in the presence of ATP has not been studied theoretically. In particular, a consistent and quantitative explanation is lacking for the available single molecule data about the dynamics of slipping of kinesin-8 in the presence of saturating ATP and that in the presence of saturating ADP and no ATP^19,20^. During the processive motion of kinesin-8, how and when the rapid slip occurs is not clear. In this work, we focus mainly on kinesin-8, studying theoretically its dynamics and addressing the unclear issues. To this end, a model is presented for the processive motion of kinesin-8 on a MT filament, on the basis of which the theoretical studies are made. The theoretical results reproduce quantitatively and consistently the prior single molecule data for kinesin-8 Kip3 about load dependences of the velocity without including the slip at saturating ATP, the velocity with including the slip at saturating ATP and the slipping velocity in the presence of saturating ADP and no ATP. Moreover, predicted results of stepping ratio at saturating ATP and moving dynamics at non-saturating ATP concentrations are provided.

## Model

**Figure 1:**
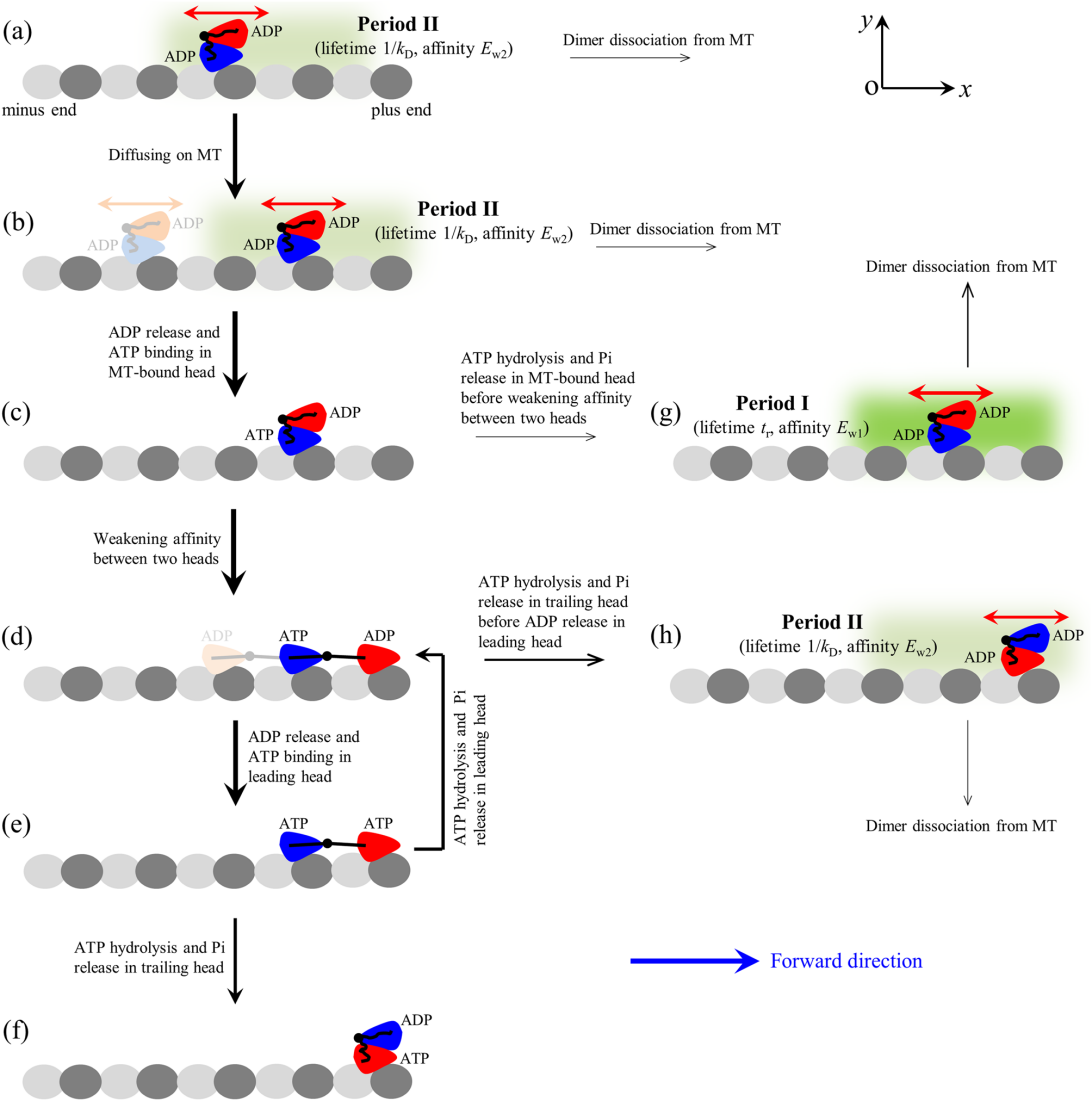
The model for processive walking and slipping of kinesin-8 motors. (**a**)–(**h**) Chemomechanical coupling pathway at saturating ATP (see text for detailed descriptions). Black arrows indicate state transitions, with the thickness of the arrow representing the magnitude of transition rate or probability under no load. Red arrows indicate slips. The blue arrow indicates the forward direction (the plus end of MT). Since in both ATP and ADP.Pi states the head has a high affinity to MT, for simplicity, we use ATP to represent both ATP and ADP.Pi states, or ATP hydrolysis and Pi release are treated as one step.

The model for processive movement of kinesin-8 motor on a MT filament powered by ATP hydrolysis is schematically shown in Fig. 1, which is modified from that proposed before for better-characterized kinesin-1 motor^32^. Before describing the model we first present the main elements based on which the model is built up.

1. In analogy to kinesin-1 head, kinesin-8 head in nucleotide-free, ATP or ADP.Pi state has a high affinity to MT whereas in ADP state has a low affinity. The high affinity has a value of *E*_s_ in the *x* direction (along MT filament) and a value of (*E*_s_ + *E*_s0_) in the *y* direction (perpendicular to MT filament) (Fig. 1), while the low affinity has a value of *E*_w2_ in the *x* direction and a value of (*E*_w2_ + *E*_w20_) in the *y* direction (noting that for kinesin-1 *E*_s0_ and *E*_w20_ were taken to be zero^32^). The presence of an extra affinity *E*_s0_ or *E*_w20_ in the *y* direction indicates a non-zero affinity between the kinesin-8 head and a MT filament at the interface between $\beta$-subunit of the previous $\alpha /\beta$-tubulin heterodimer and $\alpha$-subunit of the next tubulin heterodimer. This implies that for the potential of the interaction between the kinesin-8 head and an isolated tubulin heterodimer, the distance between the position where the potential has the minimum value and the position where the potential reaches the maximum value along the *x* direction is larger than *d*/2, with *d* being the repeat periodicity of tubulin heterodimers in a MT filament (see Section S1 and Fig. S1 in Supplementary Information for more detail). Additionally, upon Pi release the affinity of the head in ADP state to the local tubulin heterodimer, with a value of *E*_w1_ in the *x* direction and a value of (*E*_w1_ + *E*_w10_) in the *y* direction, is temporarily much lower than to other unperturbed tubulin heterodimers, i.e., *E*_w1_ + *E*_w10_ < *E*_w2_ + *E*_w20_. This can be explained as follows. The large conformational changes in the local tubulin heterodimer induced by the strong interaction with nucleotide-free, ATP- or ADP.Pi-head^33^ result in the local tubulin heterodimer having a further weaker interaction with the ADP-head than other unperturbed tubulin heterodimers, as shown by atomistic molecular dynamics (AMD) simulations for kinesin-1^34^. In a time of *t*_r_ (of the order of 10 µs), the local tubulin heterodimer relaxes to its normally unchanged conformation, with the binding energy of the local tubulin heterodimer to ADP-head becoming the same as that of other tubulin heterodimers to ADP-head. In an ATPase cycle the potential of kinesin-8 head interacting with a MT filament in the *x* direction can be schematically shown in Fig. S1 (see Supplementary Information).
2. Kinesin-8 head in ADP and nucleotide-free states possesses the conformation, with which the head has a high affinity to the other ADP-head, as AMD simulations indicated for kinesin-1^35^, and its neck linker (NL) is unable to dock onto the head, as experimental data showed for kinesin-1^36^. The head in ATP and ADP.Pi states has a large conformational change relative to that in ADP and nucleotide-free states, as the structural data showed for kineisn-1^37^. The large conformational change weakens greatly the affinity of the head to its partner ADP-head, as AMD simulations indicated for kinesin-1^35^, and makes the NL of the head able to dock, as experimental data showed for kinesin-1^37^. It is noticed however that with the NL in the backward and horizontal direction (the −*x* direction), the NL interference inhibits the large conformational change of the head in ATP and ADP.Pi states.

The chemomechanical coupling pathway at saturating ATP is described as follows. We begin with two heads in ADP state, with one head binding to MT with the low affinity of *E*_w2_ in the *x* direction and of (*E*_w2_ + *E*_w20_) in the *y* direction and the other head binding to the MT-bound head with a high affinity (Fig. 1a). By overcoming the smaller *E*_w2_ the dimer can easily move (or slip) along the *x* direction and due to relatively larger (*E*_w2_ + *E*_w20_) the dimer has a small rate to dissociate from MT (Fig. 1b). Upon ADP release from the MT-bound head, due to the high affinity *E*_s_ to MT the dimer is reluctant to slip along the *x* direction. After ATP binding to the nucleotide-free MT-bound head (Fig. 1c), the large conformational change of the head occurs rapidly, weakening greatly its affinity to the other ADP-head and inducing its NL docking. The detached ADP-head can then either diffuse forward and bind to the front MT-binding site (dark color in Fig. 1d) or diffuse backward and bind to the rear MT-binding site (light color in Fig. 1d). After the ADP-head binding to MT, ADP is released, followed by ATP binding (Fig. 1e, noting that in Fig. 1e we only show the case of the detached head binding to the front MT-binding site). In Fig. 1e, after ATP hydrolysis and Pi release in the trailing head its affinity to the local MT-binding site becomes *E*_w1_ in the *x* direction and (*E*_w1_ + *E*_w10_) in the *y* direction. Although the trailing head can now move much easier along the *x* direction than along the *y* direction, the occupancy of the next front MT-binding site by the leading head prevents the trailing head from moving to the next front MT-binding site, and the NLs of finite length prevent the trailing head from moving backward to the neighboring rear MT-binding site. However, the trailing head is allowed to move in the *y* direction, and thus within time *t*_r_ the trailing head can detach from MT by overcoming the very low affinity of (*E*_w1_ + *E*_w10_) and then diffuse to the intermediate (INT) position relative to the MT-bound head (Fig. 1f). Figure 1f is the same as Fig. 1c except that the dimer has moved forwards by one step. In Fig. 1e, ATP hydrolysis and Pi release can also occur in the leading head before in the trailing head. The leading ADP-head can detach from MT and then either bind to the rear MT-binding site next to the MT-bound ATP-head or rebind to the MT-binding site in time *t*_r_ from which the ADP-head has just detached (Fig. 1d). Note here that the movement of one head following Pi release relative to the other head for kinesin-8 is similar to that for kinesin-1 described before^32,38^.

In Fig. 1c, if ATP hydrolysis and Pi release in the MT-bound head occurs before the weakening of the affinity between the two heads, the MT-bound ADP-head has the affinity of *E*_w1_ in the *x* direction and of (*E*_w1_ + *E*_w10_) in the *y* direction to the local MT-binding site and the other detached ADP-head binds to the MT-bound head with the high affinity (Fig. 1g). The period in this state of Fig. 1g is called Period I, as done before for kinesin-1^32,39^. Then, the MT-bound head can move much easier either to the front MT-binding site or to the rear MT-binding site than detach from MT. After moving to the neighboring MT-binding site, the affinity between the ADP-head and MT becomes *E*_w2_ in the *x* direction and (*E*_w2_ + *E*_w20_) in the *y* direction. In Fig. 1d, if ATP hydrolysis and Pi release in the trailing head occur before ADP release in the leading head, the trailing head can detach from MT by overcoming affinity (*E*_w1_ + *E*_w10_) and then move to INT position relative to the other head (Fig. 1h). In Fig. 1h the MT-bound ADP-head has the affinity of *E*_w2_ in the *x* direction and of (*E*_w2_ + *E*_w20_) in the *y* direction while the other ADP-head binds to the MT-bound head with the high affinity. The period in the state of Fig. 1h is called Period II, as done before for kinesin-1^32,39^. Figure 1h is the same as Fig. 1a except that the dimer has moved forward by two steps.

## Results

### Dynamics of kinesin-8 Kip3 motor at saturating ATP

In this section, we focus on saturating ATP. We study the velocity and the forward to backward stepping ratio (simply called stepping ratio) without and with including the slips, explaining the available experimental data for the velocity and providing predicted results for the stepping ratio.

Consider that an external load *F* acts on the coiled-coil stalk of the motor, as done in the experiments^19^. Here, *F* is defined to have a positive value when it is in the forward direction. As done before for kinesin-1^32,38,40^, we consider that rate constants of the ATPase activity of the head for kinesin-8 are also independent of the force on the NL but dependent on the orientation of the NL. We denote by *k*^(+)^ and *k*^(−)^ the rate constants of ATP hydrolysis and Pi release (the rate-limiting steps of the ATPase activity) of the trailing head with forward NL orientation and the leading head with backward NL orientation, respectively. For simplicity of analysis, we treat here that the rate constant of ADP release (the non-rate-limiting step of the ATPase activity) from the MT-bound head is independent of the NL orientation. We denote by *k*_D_ the rate constant of ADP release from the MT-bound head (noting that for the ADP-head detached from MT its ADP-release rate is zero). Based on the model, the overall ATPase rates of the trailing and leading heads can be written as (see Section S3 in Supplementary Information)

1 $$ k_{{\text{T}}} = k^{( + )} P_{{\text{E}}} + \frac{{k_{{\text{D}}} k^{( + )} }}{{k_{{\text{D}}} + k^{( + )} }}\left( {1 - P_{{\text{E}}} } \right), $$

2 $$ k_{{\text{L}}} = \frac{{k_{{\text{D}}} k^{( - )} }}{{k_{{\text{D}}} + k^{( - )} }}P_{{\text{E}}} + k^{( - )} \left( {1 - P_{{\text{E}}} } \right), $$

where *P*_E_ is the probability for the detached ADP-head to move from INT position (Fig. 1c) to the front MT-binding site (dark color in Fig. 1d) and correspondingly, (1 − *P*_E_) is the probability to the rear MT-binding site (light color in Fig. 1d). In Eqs. (1) and (2) we implicitly consider that the rate constant of the reduction of the affinity between the two heads in INT state, which is equal to the rate constant of NL docking of the MT-bound ATP-head in INT state, is much larger than *k*^(+)^, *k*^(−)^ and *k*_D_. The overall ATPase rate of the motor can then be calculated by

3 $$ k = k_{{\text{T}}} + k_{{\text{L}}} . $$

The load dependence of probability *P*_E_ can be written as (see Section S3 in Supplementary Information)

4 $$ P_{{\text{E}}} = \frac{{\exp \left( {\beta E_{{{\text{NL}}}} } \right)\exp \left( {\beta Fd^{( + )} } \right)}}{{\exp \left( {\beta E_{{{\text{NL}}}} } \right)\exp \left( {\beta Fd^{( + )} } \right) + 1}}, $$

where $\beta = \left( {k_{\text{B}} T} \right)^{ - 1}$, with *k*_B_ the Boltzmann constant and *T* the absolute temperature, *E*_NL_ is the free energy change resulting from the NL docking and the large conformational change of the MT-bound head induced by ATP binding, and *d*^(+)^ is the distance parameter for the movement of the detached ADP-head from INT position to the front MT-binding site. It is simply considered that the distance parameter *d*^(−)^ for the movement of the detached ADP-head from INT position to the rear MT-binding site is approximately equal to *d*^(+)^.

The velocity of the motor without including the slip can be calculated with (see Section S3 in Supplementary Information)

5 $$ v_{{0}} = \left[ {P_{{\text{E}}} k_{{\text{T}}} - \left( {1 - P_{{\text{E}}} } \right)k_{{\text{L}}} } \right]d, $$

where *d* = 8 nm is the distance between two MT-binding sites on a MT filament. Note that *v*_0_ is defined to be positive when the motor moves forward.

It is noted that under approximation of *k*_D_ ≫ *k*^(+)^ and *k*^(−)^, Eq. (5) can be written in the simple form (see Section S3 in Supplementary Information)

6 $$ v_{0} = \frac{{r_{0}^{{\left( {1 - {F \mathord{\left/ {\vphantom {F {F_{{\text{S}}} }}} \right. \kern-\nulldelimiterspace} {F_{{\text{S}}} }}} \right)}} - 1}}{{r_{0}^{{\left( {1 - {F \mathord{\left/ {\vphantom {F {F_{{\text{S}}} }}} \right. \kern-\nulldelimiterspace} {F_{{\text{S}}} }}} \right)}} + {{k^{( + )} } \mathord{\left/ {\vphantom {{k^{( + )} } {k^{( - )} }}} \right. \kern-\nulldelimiterspace} {k^{( - )} }}}}k^{( + )} d, $$

where $r_{0} = \left( {{{k^{( + )} } \mathord{\left/ {\vphantom {{k^{( + )} } {k^{( - )} }}} \right. \kern-\nulldelimiterspace} {k^{( - )} }}} \right)\exp \left( {\beta E_{{{\text{NL}}}} } \right)$ is the stepping ratio under no load and $F_{{\text{S}}} = {{\log \left( {r_{0} } \right)} \mathord{\left/ {\vphantom {{\log \left( {r_{0} } \right)} {\left( {\beta d^{( + )} } \right)}}} \right. \kern-\nulldelimiterspace} {\left( {\beta d^{( + )} } \right)}}$ is the stall force under which *r* = 1.

Now, we present equations for the velocity resulting from the slips. We neglect the slips in the period when at least one head binds strongly to MT. Thus, the slips occur mainly in the weak MT-binding state with the MT-bound head in ADP state and the other detached head in ADP state bound with high affinity to the detached head, namely the slips occur mainly in Period I and Period II (see Fig. 1). In analogy to kinesin-1^41^, it is considered that kinesin-8 also has a much larger rate constant of NL docking than *k*^(+)^ and *k*^(−)^. Thus, the occurrence probability of Period I is much smaller than that of Period II and for a good approximation, the slips resulting from those occurring in Period I can be neglected compared to those occurring in Period II.

**Figure 2:**
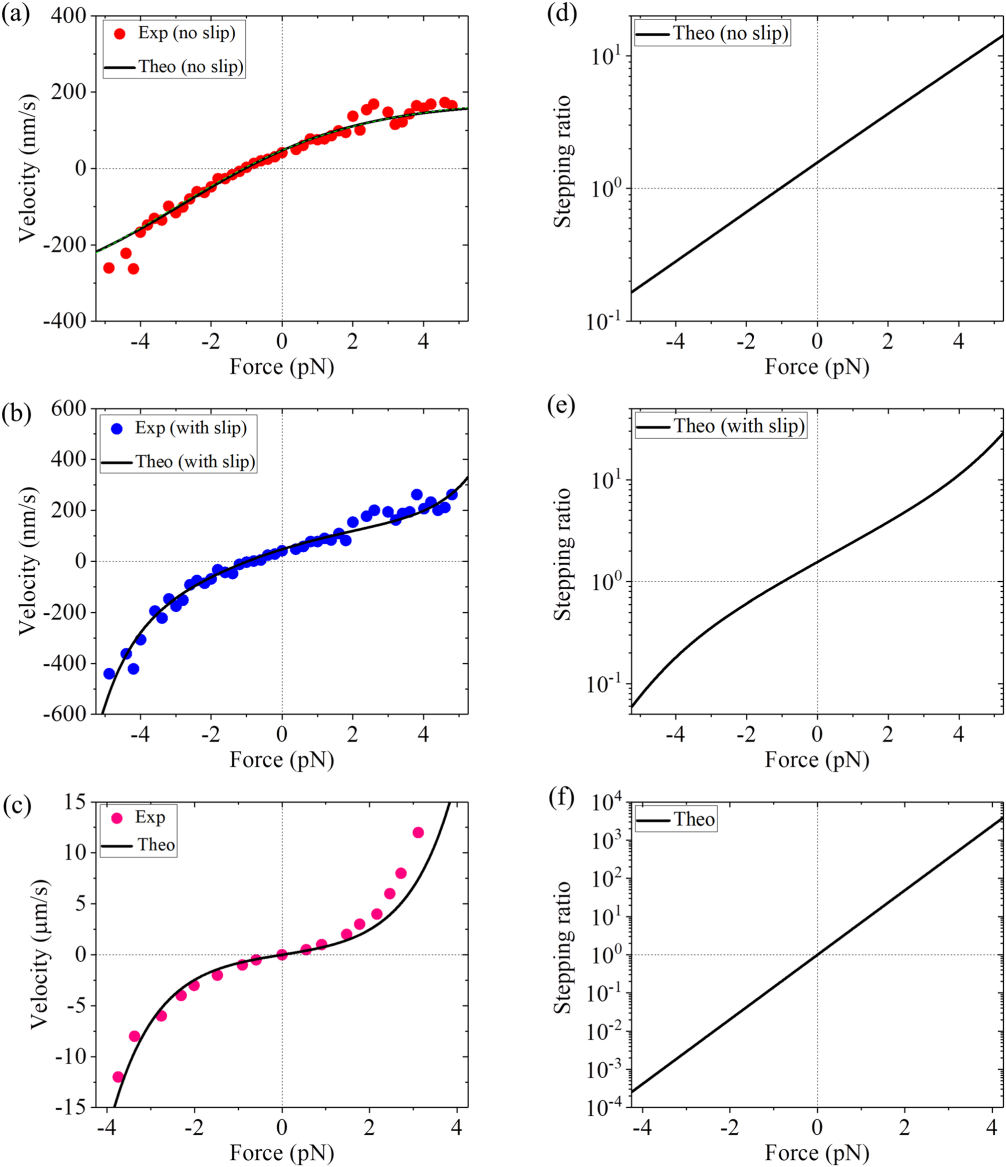
Results for dynamics of kinesin-8 Kip3 motor. (**a**) Velocity without including the slip versus load at saturating ATP. The experimental data (circles) are taken from Jannasch et al.^19^. The dashed green line, which is almost coincident with the black solid line, represents the results calculated using simple Eq. (6) and with four parameters *r*_0_ = 1.5, *F*_S_ = 1.1 pN, *k*^(+)^ = 23 ${\text{s}}^{ - 1}$ and *k*^(^^−^^)^ = 2.3*k*^(+)^. (**b**) Velocity with including the slip versus load at saturating ATP. The experimental data (circles) are taken from Jannasch et al.^19^. (**c**) Slipping velocity versus load at saturating ADP and no ATP. The experimental data (circles) are taken from Bormuth et al.^20^. (**d**) Stepping ratio without including the slip versus load at saturating ATP. (**e**) Stepping ratio with including the slip versus load at saturating ATP. (**f**) Stepping ratio versus load at saturating ADP and no ATP.

From Fig. 2, the occurrence probability of Period II in one ATPase cycle can be written as (see Section S3 in Supplementary Information)

7 $$ P_{{{\text{II}}}} = P_{{\text{E}}} \frac{{k^{( + )} }}{{k^{( + )} + k_{{\text{D}}} }} + \left( {1 - P_{{\text{E}}} } \right)\frac{{k^{( - )} }}{{k^{( - )} + k_{{\text{D}}} }}. $$

When Period II occurs, the fraction of the lifetime of Period II in the corresponding total ATPase cycle can be calculated by (see Section S3 in Supplementary Information)

8 $$ F_{{{\text{II}}}} = P_{{\text{E}}} \frac{{k^{( + )} }}{{k^{( + )} + k_{{\text{D}}} }} + \left( {1 - P_{{\text{E}}} } \right)\frac{{k^{( - )} }}{{k^{( - )} + k_{{\text{D}}} }}. $$

Assuming simply that in Period II the potential of the weak interaction between ADP-head and a tubulin heterodimer is symmetrical with respect to the loading direction, the slipping velocity in Period II as a function of external load *F* can be written as

9 $$ v_{{{\text{II}}}} = v_{{{\text{II}}}}^{(0)} \left[ {\exp \left( {\beta F\delta } \right) - \exp \left( { - \beta F\delta } \right)} \right]d, $$

where $\delta$ = *d*/2 and $v_{{{\text{II}}}}^{(0)}$ corresponds to the slipping rate under *F* = 0. As in Eq. (4), *v*_II_ is defined to be positive when the motor moves forward. It is noted here that the use of the symmetrical weak-interaction potential implies that only one unknown parameter $v_{{{\text{II}}}}^{(0)}$ is required in Eq. (9) whereas the use of the asymmetrical weak-interaction potential would require two unknown parameters. The relation between $v_{{{\text{II}}}}^{(0)}$ and the diffusion constant, *D*, can be approximately described by equations $v_{{{\text{II}}}}^{(0)} = {d \mathord{\left/ {\vphantom {d {t_{0} }}} \right. \kern-\nulldelimiterspace} {t_{0} }}$ and $d^{2} = 2Dt_{0}$, where *t*_0_ is the diffusion time per step of size *d*, giving $D = {{v_{{{\text{II}}}}^{(0)} d} \mathord{\left/ {\vphantom {{v_{{{\text{II}}}}^{(0)} d} 2}} \right. \kern-\nulldelimiterspace} 2}$.

The total velocity of the motor with including the slip can then be calculated by

10 $$ v = v_{0} + P_{{{\text{II}}}} F_{{{\text{II}}}} v_{{{\text{II}}}} . $$

In the experiments, the short slipping distances (< 16 nm) cannot be measured, which correspond usually to the short slipping times. Thus, considering that only the long slipping times were included to calculate statistically the mean slipping time, the real mean slipping time is expected to be smaller than the mean slipping time measured experimentally. Thus, we take the mean slipping time $\tau_{{\text{S}}}$ = 2.5 ms, which is half of the measured mean slipping time of about 5 ms for Kip3^19^. In our model, the slipping time $\tau_{{\text{S}}}$ corresponds to the lifetime of ADP bound to the MT-bound head in INT state, with the latter being equal to 1/*k*_D_. Consequently, in the calculation we fix *k*_D_ = 1/$\tau_{{\text{S}}}$ = 400 ${\text{s}}^{ - 1}$ (see Table 1).

**Table 1:** Parameter values for Kip3 used in the calculation.

Parameter	Description	Value
*k*_D_	Rate of ADP release from MT-bound head	400 ${\text{s}}^{ - 1}$
*k*^(+)^	Rate of ATP hydrolysis and Pi release in trailing head	22 ${\text{s}}^{ - 1}$
*k*^(−)^	Rate of ATP hydrolysis and Pi release in leading head	45 ${\text{s}}^{ - 1}$
*E*_NL_	Energy associated with NL docking and conformational change of ATP-head	1.1*k*_B_*T*
*d*^(+)^	Distance parameter for detached head moving from INT position to MT-binding site	1.7 nm
$v_{{{\text{II}}}}^{(0)}$	Slipping rate in ADP state at *F* = 0	45 ${\text{s}}^{ - 1}$
*k*_bT_	Second-order rate of ATP binding	1 ${\mu M}^{ - 1} \,{\text{s}}^{ - 1}$

From Eqs. (1)–(5), it is seen that with fixed *k*_D_, four parameters *E*_NL_, *d*^(+)^, *k*^(+)^ and *k*^(−)^ are required to calculate velocity *v*_0_ without including the slip versus external load *F* on the motor. The four parameter values can be estimated as follows. From Eq. (4) it is noted that under the large forward load *P*_E_ approaches one while under the large backward load *P*_E_ approaches zero. Thus, considering that *k*_D_ is much larger than *k*^(+)^ and *k*^(−)^, from Eqs. (1), (2) and (5) it is noted that under the large forward load *v*_0_ is approximately equal to *k*^(+)^*d* (*d* = 8 nm) while under the large backward load *v*_0_ is approximately equal to *k*^(−)^*d*. Considering *k*_D_ ≫ *k*^(+)^ and *k*^(−)^, from Eqs. (1)–(5) it is noted that under no load the velocity approximately has the form, $v_{0} \left( {F = 0} \right) = {{\left( {\exp \left( {\beta E_{{{\text{NL}}}} } \right)k^{( + )} - k^{( - )} } \right)} \mathord{\left/ {\vphantom {{\left( {\exp \left( {\beta E_{{{\text{NL}}}} } \right)k^{( + )} - k^{( - )} } \right)} {\left( {\exp \left( {\beta E_{{{\text{NL}}}} } \right) + 1} \right)}}} \right. \kern-\nulldelimiterspace} {\left( {\exp \left( {\beta E_{{{\text{NL}}}} } \right) + 1} \right)}}$. Using this expression and with values of *k*^(+)^ and *k*^(−)^ estimated from the single molecule data of *v*_0_ under the large forward and backward loads, respectively, the value of *E*_NL_ can be estimated from the single molecule data of the unloaded velocity $v_{0} \left( {F = 0} \right)$. Considering *k*_D_ ≫ *k*^(+)^ and *k*^(−)^, from Eqs. (1)–(5) it can be easily derived that velocity *v*_0_ becomes equal to 0 when load *F* = − *F*_S_ satisfies the relation, $\exp \left( {\beta E_{{{\text{NL}}}} } \right)\exp \left( { - \beta F_{{\text{S}}} d^{( + )} } \right)k^{( + )} = k^{( - )}$, where *F*_S_ is the stall force. Using this relation and with values of *k*^(+)^, *k*^(−)^ and *E*_NL_ estimated above, the value of *d*^(+)^ can be estimated from the single molecule data of the stall force *F*_S_. By adjusting the four parameters slightly around the above estimated values, the single molecule data of velocity *v*_0_ without including the slip versus *F* measured by Jannasch et al.^19^ for Kip3 can be reproduced well, as shown in Fig. 2a, where *E*_NL_ = 1.1*k*_B_*T*, *d*^(+)^ = 1.7 nm, *k*^(+)^ = 22 ${\text{s}}^{ - 1}$ and *k*^(−)^ = 45 ${\text{s}}^{ - 1}$ (see Table 1). It is interestingly noted that the very small value of *E*_NL_ = 1.1*k*_B_*T* is consistent with the experimentally measured free energy change associated with the NL docking being smaller than 1*k*_B_*T*^42^ and the AMD simulated free energy change associated with the large conformational change of the head induced by ATP binding being only about 1*k*_B_*T*^43^ for kinesin-1.

From Eqs. (1)–(10), it is seen that to calculate the velocity *v* including the slip versus *F*, besides parameters *k*_D_, *E*_NL_, *d*^(+)^, *k*^(+)^ and *k*^(−)^ another parameter $v_{{{\text{II}}}}^{(0)}$ is required, which is taken as adjustable. With above parameter values for *k*_D_, *E*_NL_, *d*^(+)^, *k*^(+)^ and *k*^(−)^, taking $v_{{{\text{II}}}}^{(0)}$ = 45 ${\text{s}}^{ - 1}$ (see Table 1) the single molecule data of the velocity *v* with including the slip versus *F* measured by Jannasch et al.^19^ for Kip3 can be reproduced quantitatively (Fig. 2b). In our model, the slipping velocity in Period II, which is calculated with Eq. (9), corresponds to the slipping velocity in the presence of saturating ADP and no ATP. With the above parameter for $v_{{{\text{II}}}}^{(0)}$, which implies the absence of any adjustable parameter, the calculated results of slipping velocity versus *F* are also in agreement with the single molecule data of Bormuth et al.^20^ for Kip3 in the presence of ADP (1 mM) and no ATP (Fig. 2c).

Taken together, with the same five adjustable parameters *E*_NL_, *d*^(+)^, *k*^(+)^, *k*^(−)^ and $v_{{{\text{II}}}}^{(0)}$, the available single molecule data on the load dependence of velocity without including the slip, velocity with including the slip and slipping velocity in the presence of saturating ADP can be reproduced well (Fig. 2a–c). The sensitivity of the theoretical results to the variation of the adjustable parameter value is also studied (see Section S3 and Figs. S4–S6 in Supplementary Information). The studies indicate that the small variation of the parameter value has only a small effect on the theoretical results.

As mentioned above, the rate constant of ADP release from the MT-bound head is independent of the force on the NL. Since the slipping time corresponds to the inverse of rate constant of ADP release from the MT-bound head in INT state, the slipping time should be independent of the load, which is consistent with the single molecule results^19^. In fact, like the rate of ATP hydrolysis and Pi release, the rate constant of ADP release should also be different for different NL orientations. In INT state, the forward load on the stalk drives the NL of the MT-bound head in the forward orientation while the backward load drives the NL in the backward orientation. Thus, the slipping time under the forward load and that under the backward load should be different, consistent with the single molecule data^19^. Since the difference is small between the slipping time (or the rate constant of ADP release) under the forward load (or for the forward NL orientation) and that under the backward load (or for the backward NL orientation)^19^, for simplicity of analysis, we take the same value for the rate constant of ADP release for different NL orientations. In addition, the experimental data showed that the addition of ADP with concentration up to 1 mM has a very small effect on the slip time at saturating ATP (1 mM)^19^, which can be explained as follows. Denoting by *k*_bT_ and *k*_bD_ the second-order rate constants of ATP and ADP binding, respectively, the lifetime of ADP bound to the MT-bound head in INT state, i.e., the slipping time, can be written as

11 $$ \tau_{{\text{S}}} = \frac{{k_{{\text{D}}} + k_{{{\text{bD}}}} [{\text{ADP}}] + k_{{{\text{bT}}}} [{\text{ATP}}]}}{{k_{{\text{D}}} k_{{{\text{bT}}}} [{\text{ATP}}]}}, $$

where [ATP] and [ADP] represent ATP and ADP concentrations, respectively. Arguing that *k*_bT_ ≫ *k*_bD_, it is seen from Eq. (11) that at [ATP] = 1 mM, the variation of [ADP] up to 1 mM has a very small effect on the slipping time. It is predicted however that in the excess of [ADP] over [ATP], the slipping time will be evidently longer than in the absence of additional ADP.

Furthermore, we present some predicted results on the stepping ratio. In the presence of saturating ATP, the stepping ratio without including the slip can be calculated with (see Section S3 in Supplementary Information)

12 $$ r_{0} = \frac{{P_{{\text{E}}} \left[ {k^{( + )} P_{{\text{E}}} + \frac{{k_{{\text{D}}} k^{( + )} }}{{k_{{\text{D}}} + k^{( + )} }}\left( {1 - P_{{\text{E}}} } \right)} \right]}}{{\left( {1 - P_{{\text{E}}} } \right)\left[ {\frac{{k_{{\text{D}}} k^{( - )} }}{{k_{{\text{D}}} + k^{( - )} }}P_{{\text{E}}} + k^{( - )} \left( {1 - P_{{\text{E}}} } \right)} \right]}}. $$

In the presence of saturating ATP, the stepping ratio with including the slip can be calculated with (see Section S3 in Supplementary Information)

13 $$ r = \frac{{P_{{\text{E}}} \left[ {k^{( + )} P_{{\text{E}}} + \frac{{k_{{\text{D}}} k^{( + )} }}{{k_{{\text{D}}} + k^{( + )} }}\left( {1 - P_{{\text{E}}} } \right)} \right] + P_{{{\text{II}}}} F_{{{\text{II}}}} v_{{{\text{II}}}}^{(0)} \exp \left( {\beta F\delta } \right)}}{{\left( {1 - P_{{\text{E}}} } \right)\left[ {\frac{{k_{{\text{D}}} k^{( - )} }}{{k_{{\text{D}}} + k^{( - )} }}P_{{\text{E}}} + k^{( - )} \left( {1 - P_{{\text{E}}} } \right)} \right] + P_{{{\text{II}}}} F_{{{\text{II}}}} v_{{{\text{II}}}}^{(0)} \exp \left( { - \beta F\delta } \right)}}. $$

In the presence of saturating ADP and no ATP, the stepping ratio can be calculated with (see Section S3 in Supplementary Information)

14 $$ r_{{{\text{II}}}} = \exp \left( {2\beta F\delta } \right). $$

Using Eqs. (12)–(14) and with parameter values given in Table 1, the calculated results of stepping ratios *r*_0_, *r* and *r*_II_ versus *F* are shown in Fig. 2d–f. The stepping ratio *r*_0_ in the presence of saturating ATP without including the slip increases exponentially with the increase of *F* (Fig. 2d). The stepping ratio *r* in the presence of saturating ATP with including the slip does not increase exponentially with the increase of *F* (Fig. 2e). The stepping ratio in the presence of saturating ADP and no ATP increases exponentially with the increase of *F* (Fig. 2f). As expected, in the presence of saturating ADP and no ATP the stepping ratio under no load is equal to one (Fig. 2f). Interestingly, in the presence of saturating ATP both the stepping ratio without including the slip and that with including the slip under no load are only about 1.6 (Fig. 2d and e).

### Dynamics of kinesin-8 Kip3 motor at non-saturating ATP

In the above section, we studied the velocity of Kip3 without and with including the slips at saturating ATP, explaining quantitatively the available single molecule data. In this section, we study the velocity of Kip3 without and with including the slips at non-saturating ATP, providing predicted results.

For simplicity, we consider that the second-order rate constant of ATP binding to the head is independent of NL orientation and denote by *k*_bT_ the second-order rate constant of ATP binding. In addition, we neglect the dissociation of ATP after its binding. From the pathway (Fig. 1), it is noted that for the case of ATP hydrolysis and Pi release occurring in the trailing head, the overall ATPase rate of the motor can be approximately calculated by (see Section S3 in Supplementary Information)

15 $$ k_{{\text{T}}} = P_{{\text{E}}} \left( {\frac{1}{{k^{( + )} }} + \frac{1}{{k_{{{\text{bT}}}} [{\text{ATP}}]}}} \right)^{ - 1} + \left( {1 - P_{{\text{E}}} } \right)\left( {\frac{1}{{k_{{\text{D}}} }} + \frac{1}{{k_{{{\text{bT}}}} [{\text{ATP}}]}} + \frac{1}{{k^{( + )} }}} \right)^{ - 1} . $$

For the case of ATP hydrolysis and Pi release occurring in the leading head, the overall ATPase rate can be approximately calculated by (see Section S3 in Supplementary Information)

16 $$ k_{{\text{L}}} = P_{{\text{E}}} \left( {\frac{1}{{k_{{\text{D}}} }} + \frac{1}{{k_{{{\text{bT}}}} [{\text{ATP}}]}} + \frac{1}{{k^{( - )} }}} \right)^{ - 1} + \left( {1 - P_{{\text{E}}} } \right)\left( {\frac{1}{{k^{( - )} }} + \frac{1}{{k_{{{\text{bT}}}} [{\text{ATP}}]}}} \right)^{ - 1} . $$

The overall ATPase rate of the motor can be calculated by $k = k_{{\text{T}}} + k_{{\text{L}}}$, which is in the same form as Eq. (3) but with different forms for *k*_T_ and *k*_L_. The velocity of the motor without including the slip can be calculated with $v_{{0}} = \left[ {P_{{\text{E}}} k_{{\text{T}}} - \left( {1 - P_{{\text{E}}} } \right)k_{{\text{L}}} } \right]d$, which is in the same form as Eq. (5), with the same *P*_E_ described by Eq. (4) but with different forms for *k*_T_ and *k*_L_.

The occurrence probability of Period II in one ATPase cycle can still be calculated by Eq. (7). When Period II occurs, the fraction of the lifetime of Period II in the corresponding total ATPase cycle can be written as (see Section S3 in Supplementary Information)

17 $$ F_{{{\text{II}}}} = P_{{\text{E}}} \frac{{{1 \mathord{\left/ {\vphantom {1 {k_{{\text{D}}} }}} \right. \kern-\nulldelimiterspace} {k_{{\text{D}}} }}}}{{{1 \mathord{\left/ {\vphantom {1 {\left( {k_{{{\text{bT}}}} [{\text{ATP}}]} \right)}}} \right. \kern-\nulldelimiterspace} {\left( {k_{{{\text{bT}}}} [{\text{ATP}}]} \right)}} + {1 \mathord{\left/ {\vphantom {1 {k^{( + )} }}} \right. \kern-\nulldelimiterspace} {k^{( + )} }} + {1 \mathord{\left/ {\vphantom {1 {k_{{\text{D}}} }}} \right. \kern-\nulldelimiterspace} {k_{{\text{D}}} }}}} + \left( {1 - P_{{\text{E}}} } \right)\frac{{{1 \mathord{\left/ {\vphantom {1 {k_{{\text{D}}} }}} \right. \kern-\nulldelimiterspace} {k_{{\text{D}}} }}}}{{{1 \mathord{\left/ {\vphantom {1 {\left( {k_{{{\text{bT}}}} [{\text{ATP}}]} \right)}}} \right. \kern-\nulldelimiterspace} {\left( {k_{{{\text{bT}}}} [{\text{ATP}}]} \right)}} + {1 \mathord{\left/ {\vphantom {1 {k^{( - )} }}} \right. \kern-\nulldelimiterspace} {k^{( - )} }} + {1 \mathord{\left/ {\vphantom {1 {k_{{\text{D}}} }}} \right. \kern-\nulldelimiterspace} {k_{{\text{D}}} }}}}. $$

The total velocity of the motor with including the slip can be calculated by $v = v_{0} + P_{{{\text{II}}}} F_{{{\text{II}}}} v_{{{\text{II}}}}$, which is in the same form as Eq. (10) with the same *P*_II_ and *v*_II_ described by Eqs. (8) and (9), respectively, but with different forms for *v*_0_ and *F*_II_.

**Figure 3:**
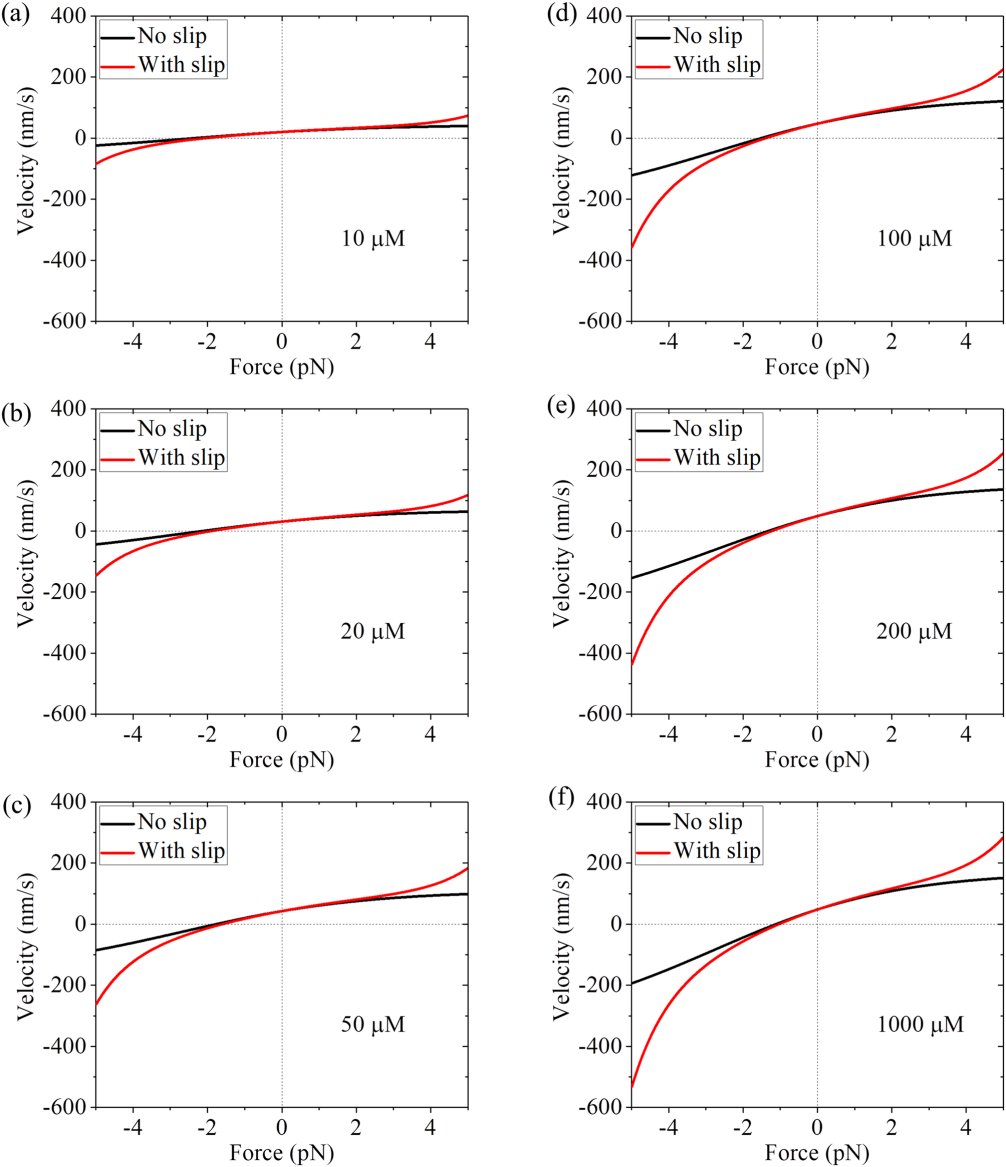
Results for velocity of kinesin-8 Kip3 motor versus load at non-saturating ATP. (**a**) 10 ${\mu M}$ ATP. (**b**) 20 ${\mu M}$ ATP. (**c**) 50 ${\mu M}$ ATP. (**d**) 100 ${\mu M}$ ATP. (**e**) 200 ${\mu M}$ ATP. (**f**) 1000 ${\mu M}$ ATP. Note that the six graphs have the same coordinate range.

**Figure 4:**
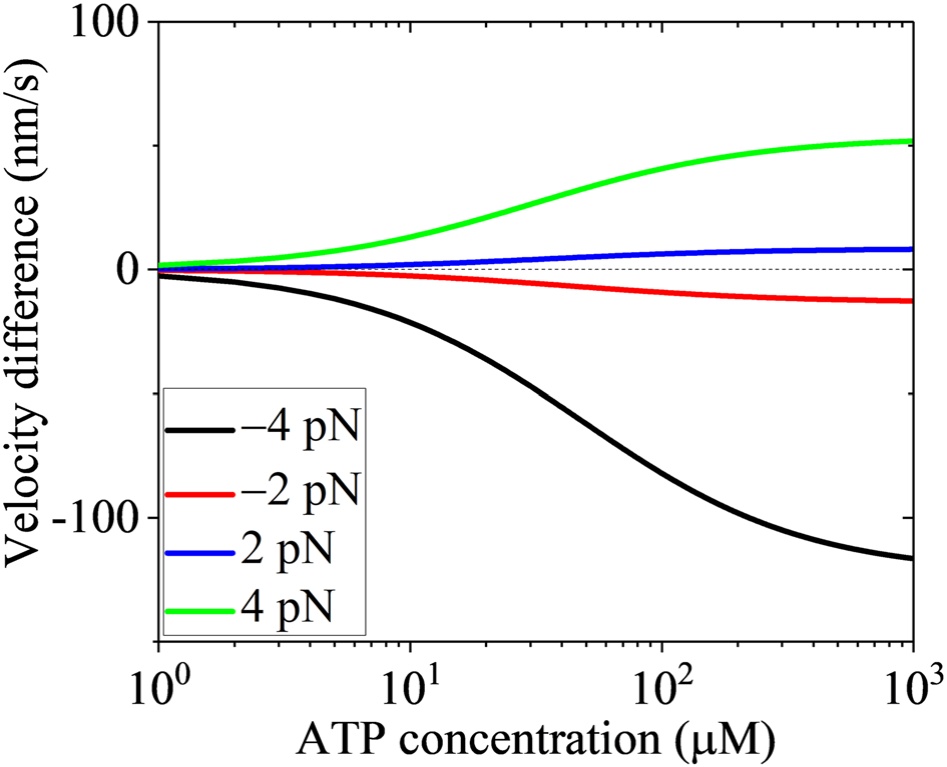
Results for the difference between the velocity with including the slip and that without including the slip (called velocity difference) of kinesin-8 Kip3 motor versus ATP concentration under different loads.

The second-order rate constant of ATP binding (*k*_bT_) for Kip3 can be estimated by comparing the theoretical results for the velocity under no load at low ATP relative to that at saturating ATP with the available corresponding experimental data. The available experimental data for Kip3 showed that the velocity under no load at 10 ${\mu M}$ ATP is about half of that at saturating ATP^44^. By taking *k*_bT_ = 1 ${\mu M}^{ - 1} \,{\text{s}}^{ - 1}$ the theoretical results of the velocity under no load at 10 ${\mu M}$ ATP is also about half of that at saturating ATP. Thus, in the calculation for Kip3 we take *k*_bT_ = 1 ${\mu M}^{ - 1} \,{\text{s}}^{ - 1}$ (see Table 1). Note that this value of *k*_bT_ for Kip3 is similar to that for kinesin-1^45^. With values of other parameters given in Table 1, we calculate the velocity for different values of *F* and ATP concentration. The results of the velocity without including the slip and that with including the slip versus *F* for different ATP concentrations are shown in Fig. 3. As expected, for a given *F* both the magnitude of the velocity without including the slip and that with including the slip increase with the increase of ATP concentration. Interestingly, the magnitude of the velocity resulting from the slip, which is equal to the difference between the velocity with including the slip and that without including the slip (simply called velocity difference), also increases with the increase of ATP concentration for a given *F*. This can also be seen clearly in Fig. 4, where we show the velocity difference versus ATP concentration for different values of *F*. From Fig. 4, it is seen that the larger the magnitude of *F* is, the larger the magnitude of the velocity difference is. Moreover, for the same magnitude of *F*, the magnitude of the velocity difference under the backward load is larger than that under the forward load.

## Discussion

### Comparison between dynamics of kinesin-8 with that of kinesin-1

As proposed here for kinesin-8 and proposed before for kinesin-1^32^, both motors use the same chemomechanical coupling mechanism except that they have different features for the interaction potential of the head with MT (see Section S1 and Fig. S1 in Supplementary Information) and different parameter values. During the normally processive movement, with at least one head binding strongly to MT, both dimers move on MT in the hand-over-hand manner (see, e.g., Fig. S2 in Supplementary Information). For both motors, the weak MT-binding state, with one head in ADP state binding weakly to MT and the other detached ADP-head binding to the MT-bound head with the high affinity, can occur occasionally (Fig. 1)^32^. During the normally processive motion without the occasional occurrence of the weak MT-binding state, since the two motors show the same stepping pathway the velocity of the two motors can be described by the same expression as described by Eq. (5)^32^. For the two motors, since the occurrence of the weak MT-binding state arises from the same chemomechanical coupling mechanism, the occurrence probability of the weak MT-binding state (or the weak MT-binding period) can be described by the same expression as described by Eq. (7)^32^.

However, due to different features for the interaction potential of the head with MT, the two motors show different dynamical behaviors in the weak MT-binding state or during the weak MT-binding period. For kinesin-8, in the weak MT-binding state, the affinity is *E*_w1_ (Period I) and *E*_w2_ (Period II) in the *x* direction while is (*E*_w1_ + *E*_w10_) (Period I) and (*E*_w2_ + *E*_w20_) (Period II) in the *y* direction. Thus, kinesin-8 motor can move much easier along the *x* direction than along the *y* direction, resulting in most probably slipping along the *x* direction and rarely dissociation along the *y* direction. By contrast, for kinesin-1, in the weak MT-binding state, the affinity is *E*_w1_ (Period I) and *E*_w2_ (Period II) in both the *x* and *y* directions, resulting in that the motor most probably dissociates from MT rather than slips along MT. Therefore, kinesin-8 motor can slip frequently and has a long run length. By contrast, kinesin-1 motor slips rarely and has a relatively short run length.

### Biological functions of kinesin-8 and kinesin-1 can be explained by the model

It is noted that for kinesin-8 the presence of extra *E*_s0_ and *E*_w20_ implies that after reaching the plus end of a MT filament the motor in one-head-bound state cannot dissociate easily from MT. In addition, the very small *E*_NL_ of 1.1*k*_B_*T* for kinesin-8 (Table 1), which results in the small stepping ratio of only about 1.6 under no load (Fig. 2d–f), implies that the detached ADP-head can diffuse easily from INT position to the rear MT-binding site. Thus, when the motor reaches MT plus end, after Pi release from the trailing head the detached ADP-head can diffuse rapidly from INT position (corresponding to one-head-bound state) to the rear MT-binding site (corresponding to two-heads-bound state) because now no front MT-binding site is present. The above two properties for kinesin-8 thus indicate that after reaching MT plus end the motor has a small rate to dissociate from the MT, ensuring the kinesin-8 to stay at the MT plus end for a long time to perform the function of depolymerizing the MT.

By contrast, for kinesin-1 the absence of extra *E*_s0_ and *E*_w20_ implies that after reaching the MT plus end the motor in one-head-bound state can dissociate easily from MT. In addition, the relatively large *E*_NL_ of about 3.5*k*_B_*T* for kinesin-1^32^, which results in the large stepping ratio of about 800 under no load^32^, implies that the detached ADP-head cannot diffuse easily from INT position to the rear MT-binding site. Thus, when the motor reaches MT plus end, after Pi release from the trailing head the detached ADP-head cannot move easily from INT position (corresponding to one-head-bound state) to the rear MT-binding site (corresponding to two-heads-bound state). The above two properties for kinesin-1 thus indicate that after reaching the MT plus end the motor has a large rate to dissociate from the MT, implying that the kinesin-1 bound by the cargo can dissociate rapidly from the MT plus end, terminating the cargo transport.

### Concluding remarks

A model is proposed for the chemomechanical coupling of kinesin-8 Kip3 motor (Fig. 1), showing that the motor moves processively on MT in the hand-over-hand manner and the movement is interrupted frequently by brief and rapid slips. On the basis of the model, the dynamics of Kip3 motor is studied analytically. The analytical results explain quantitatively the available single molecule data on velocity without including the slip and that with including the slip versus external load at saturating ATP. The analytical results also explain well the available single molecule data on slipping velocity of the motor versus external load at saturating ADP and no ATP. Moreover, the predicted results of stepping ratio versus the external load at saturating ATP and at saturating ADP and no ATP are also provided. The predicted results are also provided for velocity without including the slip and that with including the slip versus external load at various ATP concentrations. These predicted results can be tested easily using single molecule optical trapping methods.

As shown elsewhere^32,39,40,46^, with the similar chemomechanical coupling pathway to Fig. 1 diverse available experimental data on dynamics of kinesin-1 such as the load dependences of velocity, stepping ratio, run length, dissociation rate, etc., can be reproduced quantitatively. In addition, with the similar pathway the available experimental data for other families of N-type kinesin motors such as dimeric kinesin-2 and cargo-induced dimerized kinesin-3 can also be explained quantitatively^40,47,48^. In particular, the surprising experimental data for N-type orphan kinesin PAKRP2 dimer, showing that although its head has a NL of long length (32 residues) it can also walk processively on MT in a hand-over-hand manner with one step per ATP, can also be explained quantitatively^49^. Thus, it is expected that all of the N-type kinesin dimers could use the similar chemomechanical coupling mechanism for their processive motility.

## Supplementary Information

Supplementary Information.
