# Supplementary material for: A model of processive walking and slipping of kinesin-8 molecular motors
Source: Sci Rep. 2021 Apr 13;11:8081. doi: 10.1038/s41598-021-87532-0 (PMC8044202; doi:10.1038/s41598-021-87532-0)
Supplement: Supplementary file 1 — Supplementary Information. [file 41598_2021_87532_MOESM1_ESM.pdf]

Supplementary Information

A model of processive walking and slipping of kinesin-8 molecular motors

Ping Xie

Key Laboratory of Soft Matter Physics, Institute of Physics, Chinese Academy of Sciences, Beijing 100190, China

S1. Interaction potential between kinesin head and MT

Fig. S1a shows schematically the interaction potential between a kinesin-8 head in ADP state and an isolated α / β-tubulin heterodimer in the x direction, where the distance between the position where the potential has the minimum value and the position where the potential reaches the maximum value along the x direction is larger than $d/2$, with d being the repeat periodicity of tubulin heterodimers in a MT filament. Fig. S1b shows schematically the interaction potential of a kinesin-8 head in ADP state with a MT filament in the x direction or along the filament. For comparison, in Fig. S1c we show schematically the interaction potential between a kinesin-1 head in ADP state and an isolated α / β-tubulin heterodimer in the x direction, where the distance between the position where the potential has the minimum value and the position where the potential reaches the maximum value along the x direction is smaller than or equal to $d/2$. In Fig. S1d we show schematically the interaction potential of a kinesin-1 head in ADP state with a MT filament in the x direction or along the filament.

Fig. S1e – g shows schematically the interaction potential between a kinesin-8 head during an ATPase cycle and a MT filament in the x direction. Fig. S1e corresponds to the kinesin-8 head in nucleotide-free (ϕ), ATP and ADP.Pi states interacting strongly with MT. The strong interaction induces large conformational changes of the local α / β-tubulin heterodimer, as prior structural data showed [S1]. Fig. S1f corresponds to the kinesin-8 head interacting weakly with MT immediately after Pi release, where the head has a much weaker affinity E_{w1} in the x direction to

the local α / β -tubulin heterodimer with the large conformational changes than affinity E_{w1} to other α / β -tubulin heterodimer without the conformational changes, as prior all-atom molecular dynamics showed [S2]. In a time of t_r (of the order of 10 μs), the local α / β -tubulin heterodimer relaxes to its normally unchanged conformation, with the binding energy of the local tubulin heterodimer to ADP-head becoming the same as that of other tubulin heterodimers to ADP-head. Thus, the interaction potential can be shown schematically in Fig. S1g. After ADP release, the interaction potential returns to that shown in Fig. S1e.

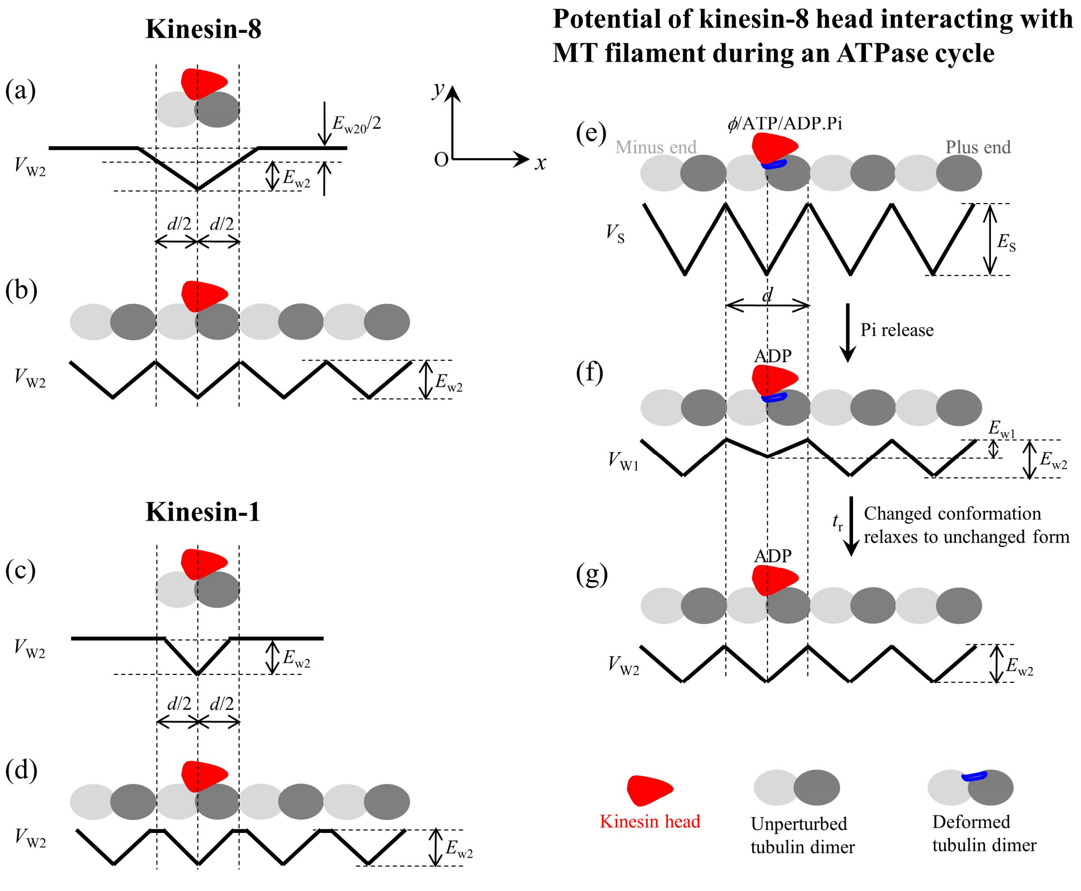

Figure S1. Schematic illustrations of the interaction potential of a kinesin head with MT. **(a)** Potential of kinesin-8 head in ADP state interacting with an isolated α / β -tubulin heterodimer in the x direction. **(b)** Potential of kinesin-8 head in ADP state interacting with a MT filament in the x direction. **(c)** Potential of kinesin-1 head in ADP state interacting with an isolated α / β -tubulin heterodimer in the x

direction. **(d)** Potential of kinesin-1 head in ADP state interacting with a MT filament in the x direction. **(e – g)** Potential of kinesin-8 head interacting with a MT filament in the x direction during an ATPase cycle.

S2. Chemomechanical coupling pathway without including the occurrence of weak-binding Period I and Period II

The detailed chemomechanical coupling pathway without consideration of the occurrence of weak-binding Period I and Period II at low ATP concentrations is schematically shown in Fig. S2. Let us start with the trailing head in ATP state binding strongly to site II on a MT filament and the leading head in ADP state binding to site III (Fig. S2a). Stimulated by MT, ADP is released rapidly from the leading head (Fig. S2b). After ATP hydrolysis and Pi release takes place in the trailing head, the head diffuses rapidly to INT position relative to the MT-bound ϕ-head, where the two heads have a high affinity (Fig. S2c). After ATP binding to the MT-bound ϕ-head (Fig. S2d), the large conformational change of the head takes place rapidly, weakening greatly its affinity to the other ADP-head and inducing its NL docking (Fig. S2e). The detached ADP-head then diffuses rapidly either (with probability P_E) to the front site IV (Fig. S2f) or (with probability $1–P_E$) to the rear site II (Fig. S2g). Stimulated by MT, ADP is released rapidly from the trailing head (Fig. S2h). After ATP hydrolysis and Pi release takes place in the leading head, the head diffuses rapidly to INT position relative to the MT-bound ϕ-head, where the two heads have the high affinity (Fig. S2i). After ATP binding to the MT-bound ϕ-head (Fig. S2j), the large conformational change of the head takes place rapidly, weakening greatly its affinity to the ADP-head and inducing its NL docking (Fig. S2k). The detached ADP-head then diffuses rapidly either (with probability P_E) to the front site III (Fig. S2a) or (with probability $1–P_E$) to the rear site I (Fig. S2l). Additionally, in Fig. S2b before ATP hydrolysis and Pi release taking place in the trailing head ATP can also bind to the leading head (Fig. S2m). Similarly, in Fig. S2h before ATP hydrolysis and Pi release taking place in the leading head ATP can also bind to the trailing ϕ-head (Fig. S2m). In Fig. 2m, if ATP hydrolysis and Pi release take place in the trailing head the system becomes the state of Fig. S2d, while if ATP hydrolysis and Pi release take place in the leading head the system becomes the state of Fig. S2k.

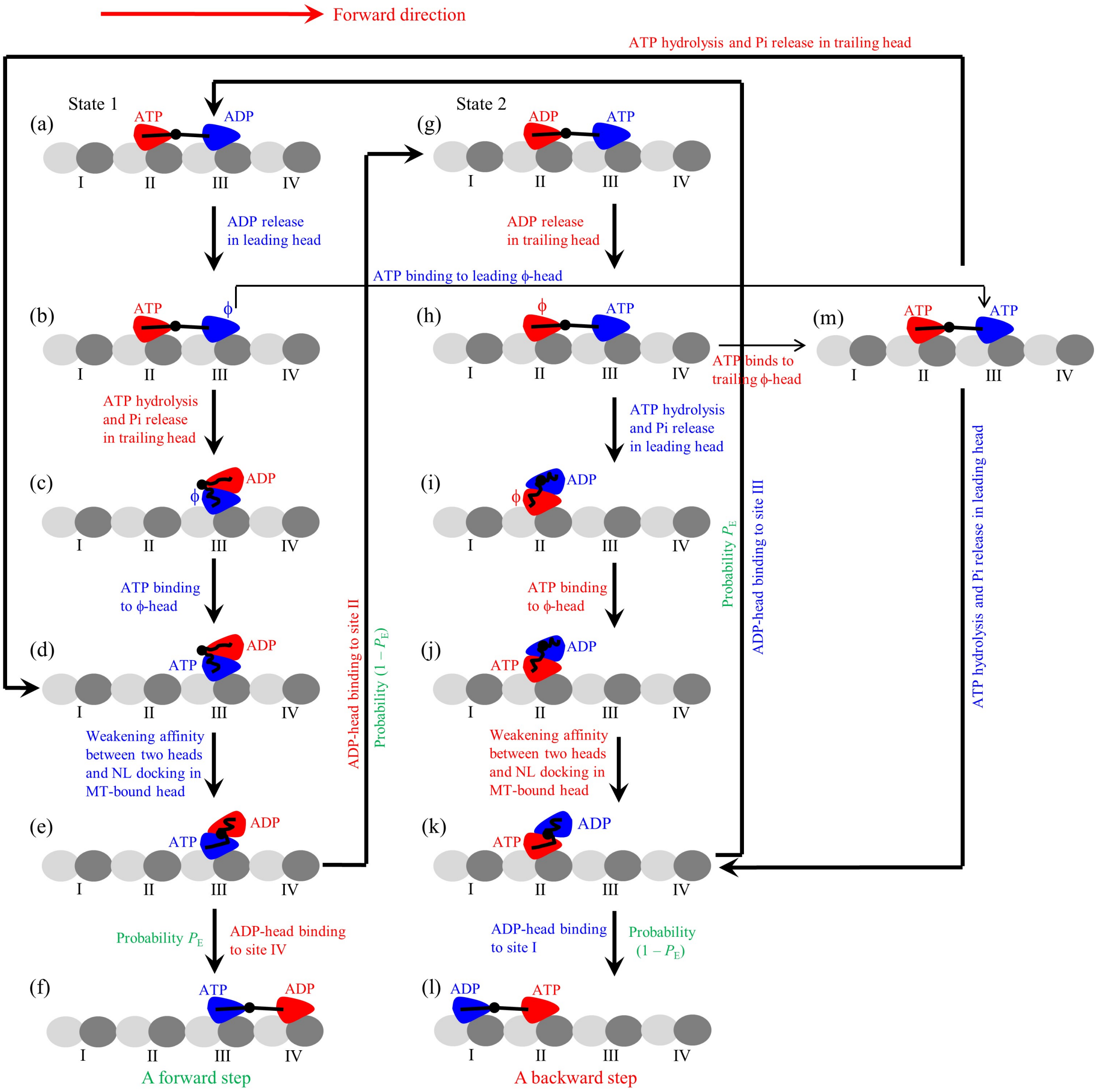

Figure S2. Schematic illustrations of the stepping of kinesin dimer at low ATP concentrations. **(a)** – **(m)** The pathway of chemomechanical coupling of kinesin dimer without consideration of the occurrence of weak-binding Period I and Period II. For simplicity, ATP hydrolysis and Pi release are treated here as one step, with symbol ATP representing both ATP and ADP.Pi states, because in both ATP and ADP.Pi states the head binds strongly to MT. As a result, the transition from ATP to ADP shown here is composed of both the transition of ATP to ADP.Pi and that of ADP.Pi to ADP.

S3. Expressions for load dependence of kinesin-8 dynamics

First, we derive the equation for load dependence of effective probability P_{E} (defined in Fig. S2). Since NL is flexible, it is considered that NL cannot bear the compressive force acting on its two ends and can only bear the stretching force. Thus, during the stepping period when only one head is bound to MT it is approximately considered that a backward load ($F < 0$) on the stalk approximately acts only on the kinesin head in the leading position while a forward load ($F > 0$) approximately acts only on the head in the trailing position. As a result, after the trailing head releases Pi the backward load ($F < 0$) has no effect on its movement to INT position and after the affinity between the two heads becomes weak the backward load ($F < 0$) has no effect on the movement of the detached ADP-head from INT position to the rear MT-binding site. After the affinity between the two heads becomes weak, under the backward load ($F < 0$) the rate for the detached ADP-head to move from INT position to the front MT-binding site can be written as $k_{\mathrm{F}} = C\exp\left(\beta F d^{(+)}\right)$, where C is a constant independent of F, $d^{(+)}$ is the distance parameter for the movement of the detached head from INT position to the front MT-binding site, and $\beta^{-1} = k_{\mathrm{B}}T$, with k_{B} being the Boltzmann constant and T being the absolute temperature. Under the backward load ($F < 0$) the rate for the detached ADP-head to move from INT position to the rear MT-binding site can be written as $k_{\mathrm{R}} = C\exp\left(-\beta E_{\mathrm{NL}}\right)$, where E_{NL} is the energy change associated with both the NL docking and the conformational change of the head induced by ATP binding [S3]. The effective probability P_{E} can be calculated with $P_{\mathrm{E}} = k_{\mathrm{F}}/\left(k_{\mathrm{F}} + k_{\mathrm{R}}\right)$, which can be rewritten as

$$P_{\mathrm{E}} = \frac{\exp\left(\beta E_{\mathrm{NL}}\right)\exp\left(\beta F d^{(+)}\right)}{\exp\left(\beta E_{\mathrm{NL}}\right)\exp\left(\beta F d^{(+)}\right) + 1}. \tag{S1}$$

Similarly, under a forward load ($F > 0$) the effective probability P_{E} can still be written in the form of Eq. (S1), but with $d^{(+)}$ being replaced with $d^{(-)}$, where $d^{(-)}$ is the distance parameter for the movement of the detached head from INT position to the rear MT-binding site. Under the approximation of $d^{(+)} = d^{(-)}$, it is seen that under both backward and forward loads, P_{E} has the same form.

Second, we derive equations for the ATPase rate of the dimer during its processive movement at saturating ATP. From Fig. S2, it is noted that during the processive movement the state of the dimer with the trailing head in ATP state and the

leading head in ADP state (call State 1) occurs with probability P_E while the state of the dimer with the trailing head in ADP state and the leading head in ATP state (called State 2) occurs with probability $1-P_E$. In State 1 the rate of Pi release occurring in the trailing head is $k^{(+)}$ and in State 2 the rate of Pi release occurring in the trailing head is $\left(1/k_D + 1/k^{(+)}\right)^{-1}$. Since ADP release is not rate limiting, with $k_D >> k^{(+)}$ and $k_D >> k^{(-)}$, it is approximately considered that ADP release from the leading head in State 1 occurs before Pi release in the trailing head and ADP release from the trailing head in State 2 occurs before Pi release in the leading head. Considering that the rate of NL docking or the rate of weakening affinity between the two heads in INT state is much larger than rate constants of ATPase activity, the overall ATPase rate of the trailing head can be approximately calculated with

$$k_T = k^{(+)} P_E + \frac{k_D k^{(+)}}{k_D + k^{(+)}} \left(1 - P_E\right). \tag{S2}$$

In State 1 the rate of Pi release occurring in the leading head is $\left(1/k_D + 1/k^{(-)}\right)^{-1}$ and in State 2 the rate of Pi release occurring in the leading head is $k^{(-)}$. Thus, the overall ATPase rate of the leading head can be approximately calculated with

$$k_L = \frac{k_D k^{(-)}}{k_D + k^{(-)}} P_E + k^{(-)} \left(1 - P_E\right). \tag{S3}$$

Therefore, from Fig. S2 it is noted that the velocity of the motor without consideration of the occurrence of the weak-binding Period I and Period II (or without including the slip) can be calculated with

$$v_0 = \left[P_E k_T - \left(1 - P_E\right) k_L\right] d\,, \tag{S4}$$

where $d = 8$ nm is the distance between two MT-binding sites on a MT filament. Note that v_0 is defined to be positive when the motor moves forward.

It is noted that under approximation of $k_D >> k^{(+)}$ and $k^{(-)}$, from Eqs. (S2) and (S3) we have $k_T = k^{(+)}$ and $k_L = k^{(-)}$. Substituting above k_T and k_L and Eq. (S1) into Eq. (S4) we obtain

$$v_0 = \left(\frac{\frac{k^{(+)}}{k^{(-)}} \exp\left(\beta E_{NL}\right) \exp\left(\beta F d^{(+)}\right) - 1}{\frac{k^{(+)}}{k^{(-)}} \exp\left(\beta E_{NL}\right) \exp\left(\beta F d^{(+)}\right) + \frac{k^{(+)}}{k^{(-)}}} \right) k^{(+)} d\,. \tag{S5}$$

By defining $r_0 = \left(k^{(+)}/k^{(-)}\right) \exp\left(\beta E_{NL}\right)$ and $F_S = \log\left(r_0\right) / \left(\beta d^{(+)}\right)$, Eq. (S5) can be

rewritten as

$$v_0 = \frac{r_0^{(1-F/F_S)} - 1}{r_0^{(1-F/F_S)} + k^{(+)}/k^{(-)}} k^{(+)} d \,. \tag{S6}$$

Now, we consider the occasional occurrence of Period II in one ATPase cycle. In the model (see Fig. S2), Period II comes from two states—State 1 and State 2. In State 1, if ATP hydrolysis and Pi release in the trailing head take place before ADP release from the leading head Period II occurs (transition from Fig. S3a to b). In State 2, if ATP hydrolysis and Pi release in the leading head take place before ADP release from the trailing head Period II occurs (transition from Fig. S3c to d). In one ATPase cycle, the occurrence probability of transition from Fig. S3a to b can be calculated by $P_E\, k^{(+)} \big/ \left(k^{(+)} + k_D\right)$ while the occurrence probability of transition from Fig. S3c to d can be calculated by $\left(1 - P_E\right) k^{(-)} \big/ \left(k^{(-)} + k_D\right)$. Thus, the occurrence probability of Period II in one ATPase cycle can be calculated by

$$P_{II} = P_E \frac{k^{(+)}}{k^{(+)} + k_D} + \left(1 - P_E\right) \frac{k^{(-)}}{k^{(-)} + k_D} \,. \tag{S7}$$

When Period II occurs, the lifetime of Period II is $1/k_D$. Thus, at saturating ATP, if Period II comes from State 1 the fraction of the lifetime of Period II in the corresponding ATPase cycle is $1/k_D \big/ \left(1/k^{(+)} + 1/k_D\right)$ and if Period II comes from State 2 the fraction of the lifetime of Period II in the corresponding ATPase cycle is $1/k_D \big/ \left(1/k^{(-)} + 1/k_D\right)$. Consequently, at saturating ATP, when Period II occurs the fraction of the lifetime of Period II in the corresponding total ATPase cycle at saturating ATP can be calculated by

$$F_{II} = P_E \frac{1/k_D}{1/k^{(+)} + 1/k_D} + \left(1 - P_E\right) \frac{1/k_D}{1/k^{(-)} + 1/k_D} \,. \tag{S8}$$

Eq. (S8) can be rewritten as

$$F_{II} = P_E \frac{k^{(+)}}{k^{(+)} + k_D} + \left(1 - P_E\right) \frac{k^{(-)}}{k^{(-)} + k_D} \,. \tag{S9}$$

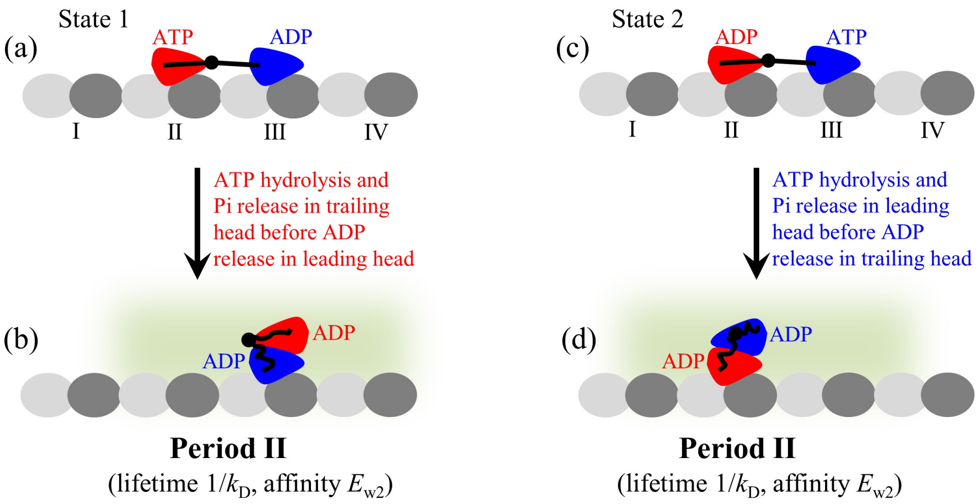

Figure S3. Schematic of state transitions for occurrence of Period II. **(a, b)** Period II comes from State 1 with trailing head in ATP state and leading head in ADP state. **(c, d)** Period II comes from State 2 with trailing head in ADP state and leading head in ATP state.

In the presence of saturating ATP, without including the slip the forward stepping rate is $P_E k_T$ and the backward stepping rate is $(1-P_E)k_L$. Thus, the stepping ratio without including the slip can be calculated by $r_0 = P_E k_T / [(1-P_E)k_L]$. Substituting Eqs. (S2) and (S3) into above equation, we obtain

$$r_0 = \frac{P_E\left[k^{(+)}P_E + \dfrac{k_D k^{(+)}}{k_D + k^{(+)}}(1-P_E)\right]}{(1-P_E)\left[\dfrac{k_D k^{(-)}}{k_D + k^{(-)}}P_E + k^{(-)}(1-P_E)\right]}. \tag{S10}$$

As seen in Eq. (9) (see main text), in the presence of saturating ADP and no ATP the forward stepping rate is $v_{II}^{(0)}\exp(\beta F\delta)$ and the backward stepping rate is $v_{II}^{(0)}\exp(-\beta F\delta)$. Thus, the stepping ratio at saturating ADP and no ATP can be calculated with $r_{II} = v_{II}^{(0)}\exp(\beta F\delta) / \left[v_{II}^{(0)}\exp(-\beta F\delta)\right]$, which can be rewritten as

$$r_{II} = \exp(2\beta F\delta). \tag{S11}$$

At saturating ATP, with including the slip the forward stepping rate is $P_E k_T + P_{II} F_{II} v_{II}^{(0)}\exp(\beta F\delta)$ and the backward stepping rate is

$\left(1-P_{\mathrm{E}}\right)k_{\mathrm{L}}+P_{\mathrm{II}}F_{\mathrm{II}}v_{\mathrm{II}}^{(0)}\exp\left(-\beta F\delta\right)$. Thus, the stepping ratio with including the slip can be calculated with $r_0=\left[P_{\mathrm{E}}k_{\mathrm{T}}+P_{\mathrm{II}}F_{\mathrm{II}}v_{\mathrm{II}}^{(0)}\exp\left(\beta F\delta\right)\right]\Big/\left[\left(1-P_{\mathrm{E}}\right)k_{\mathrm{L}}+P_{\mathrm{II}}F_{\mathrm{II}}v_{\mathrm{II}}^{(0)}\exp\left(-\beta F\delta\right)\right]$. Substituting Eqs. (S2) and (S3) into above equation, we obtain

$$r=\frac{P_{\mathrm{E}}\left[k^{(+)}P_{\mathrm{E}}+\dfrac{k_{\mathrm{D}}k^{(+)}}{k_{\mathrm{D}}+k^{(+)}}\left(1-P_{\mathrm{E}}\right)\right]+P_{\mathrm{II}}F_{\mathrm{II}}v_{\mathrm{II}}^{(0)}\exp\left(\beta F\delta\right)}{\left(1-P_{\mathrm{E}}\right)\left[\dfrac{k_{\mathrm{D}}k^{(-)}}{k_{\mathrm{D}}+k^{(-)}}P_{\mathrm{E}}+k^{(-)}\left(1-P_{\mathrm{E}}\right)\right]+P_{\mathrm{II}}F_{\mathrm{II}}v_{\mathrm{II}}^{(0)}\exp\left(-\beta F\delta\right)}. \tag{S12}$$

Up to now, we have focused on saturating ATP concentrations. Now, we consider low ATP concentrations. It is evident that the effective probability P_{E} is independent of the ATP concentration. Thus, at low ATP concentrations, the load dependence of P_{E} still has the form of Eq. (S1).

At low ATP concentrations the equations for the ATPase rate of the dimer during its processive movement can be derived as follows. For the case of ATP hydrolysis and Pi release occurring in the trailing head, from State 1 the ATPase rate can be approximately calculated by $\left[1/k^{(+)}+1/\left(k_{\mathrm{bT}}[\mathrm{ATP}]\right)\right]^{-1}$. From State 2 the ATPase rate can be calculated by $\left[1/k_{\mathrm{D}}+1/\left(k_{\mathrm{bT}}[\mathrm{ATP}]\right)+1/k^{(+)}\right]^{-1}$. Thus, for the case of ATP hydrolysis and Pi release occurring in the trailing head, the overall ATPase rate can be approximately calculated by

$$k_{\mathrm{T}}=P_{\mathrm{E}}\left(\frac{1}{k^{(+)}}+\frac{1}{k_{\mathrm{bT}}[\mathrm{ATP}]}\right)^{-1}+\left(1-P_{\mathrm{E}}\right)\left(\frac{1}{k_{\mathrm{D}}}+\frac{1}{k_{\mathrm{bT}}[\mathrm{ATP}]}+\frac{1}{k^{(+)}}\right)^{-1}. \tag{S13}$$

Similarly, for the case of ATP hydrolysis and Pi release occurring in the leading head, the overall ATPase rate can be approximately calculated by

$$k_{\mathrm{L}}=P_{\mathrm{E}}\left(\frac{1}{k_{\mathrm{D}}}+\frac{1}{k_{\mathrm{bT}}[\mathrm{ATP}]}+\frac{1}{k^{(-)}}\right)^{-1}+\left(1-P_{\mathrm{E}}\right)\left(\frac{1}{k^{(-)}}+\frac{1}{k_{\mathrm{bT}}[\mathrm{ATP}]}\right)^{-1}. \tag{S14}$$

It is noted that the occurrence probability of Period II in one ATPase cycle is independent of the ATP concentration. Thus, at low ATP concentrations, P_{II} still has the form of Eq. (S7).

At low ATP concentrations, if Period II comes from State 1 the fraction of the lifetime of Period II in the corresponding ATPase cycle is $1/k_{\mathrm{D}}\Big/\left(1/k^{(+)}+1/\left(k_{\mathrm{bT}}[\mathrm{ATP}]\right)+1/k_{\mathrm{D}}\right)$ and if Period II comes from State 2 the fraction of

the lifetime of Period II in the corresponding ATPase cycle is $1/k_{\rm D}\Big/\left(1/k^{(-)}+1/\left(k_{\rm bT}[{\rm ATP}]\right)+1/k_{\rm D}\right)$. Consequently, at low ATP concentrations, when Period II occurs the fraction of the lifetime of Period II in the corresponding total ATPase cycle can be calculated by

$$F_{\rm II}=P_{\rm E}\frac{1/k_{\rm D}}{1/\left(k_{\rm bT}[{\rm ATP}]\right)+1/k^{(+)}+1/k_{\rm D}}+\left(1-P_{\rm E}\right)\frac{1/k_{\rm D}}{1/\left(k_{\rm bT}[{\rm ATP}]\right)+1/k^{(-)}+1/k_{\rm D}}. \quad \text{(S15)}$$

S4. Sensitivity of theoretical results to the variation of adjustable parameter value at saturating ATP

To see the sensitivity of the theoretical results to the variation of the adjustable parameter value, we study the variation of the theoretical results in terms of the variation of each value of the five adjustable parameters $E_{\rm NL}$, $d^{(+)}$, $k^{(+)}$, $k^{(-)}$ and $v_{\rm II}^{(0)}$. The results are shown in Figs. S4 – S6. It is seen that the small variation of a parameter value has only a small effect on the theoretical results. As expected, both the velocity without including the slip and that with including the slip under the forward load are sensitive to $k^{(+)}$ while under the backward load are sensitive to $k^{(-)}$. The variation of $E_{\rm NL}$ has a more sensitive effect on the velocity without including the slip and that with including the slip under the backward load than under the forward load. The variation of $d^{(+)}$ also has a more sensitive effect on the velocity without including the slip and that with including the slip under the backward load than under the forward load. The variation of $v_{\rm II}^{(0)}$ has a more sensitive effect on the velocity with including the slip and the slipping velocity at saturating ADP under large forward and backward loads.

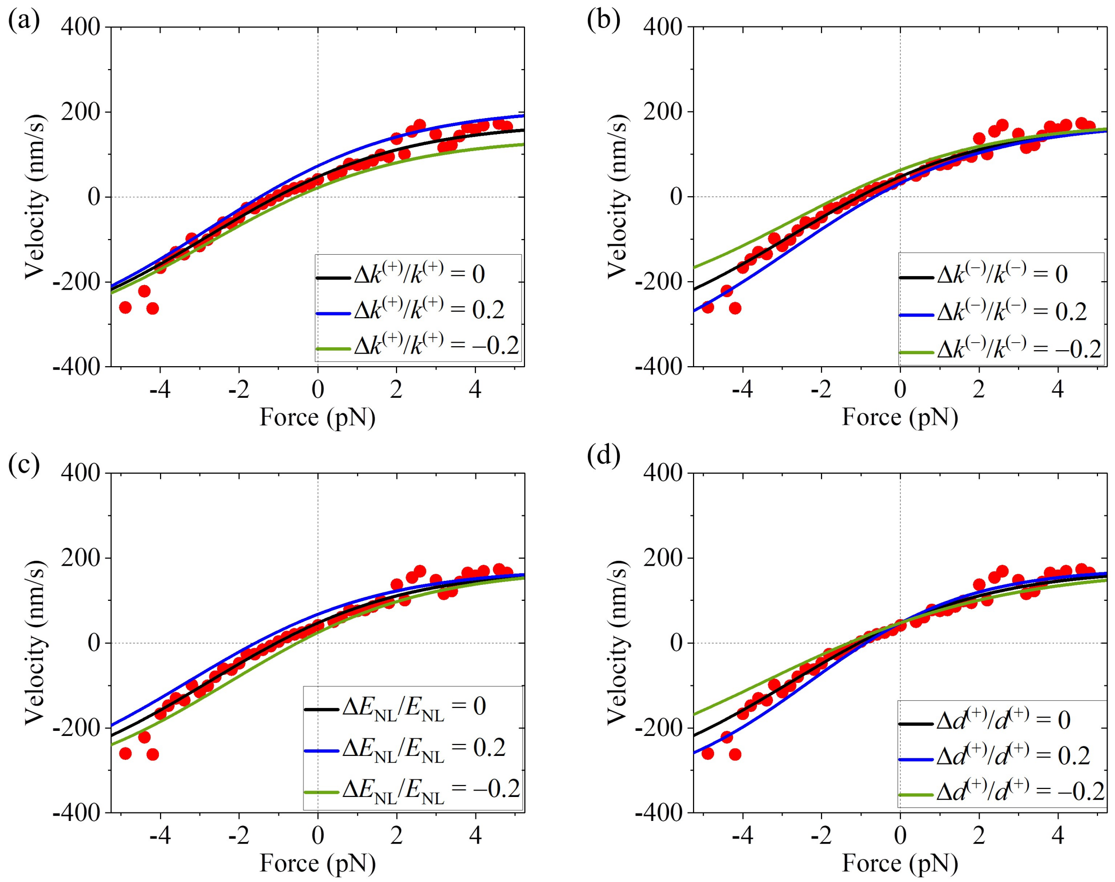

Figure S4. The sensitivity of theoretical results for the load dependence of velocity without including the slip to the variation of the adjustable parameter at saturating ATP. Lines are theoretical results calculated with parameter values listed in Table 1 except that one parameter value is changed. The symbols are experimental data taken from Jannasch et al. [S4]. **(a)** Effect of variation of individual $k^{(+)}$ by 20% on the results. **(b)** Effect of variation of individual $k^{(-)}$ by 20% on the results. **(c)** Effect of variation of individual E_{NL} by 10% on the results. **(d)** Effect of variation of individual $d^{(+)}$ by 20% on the results.

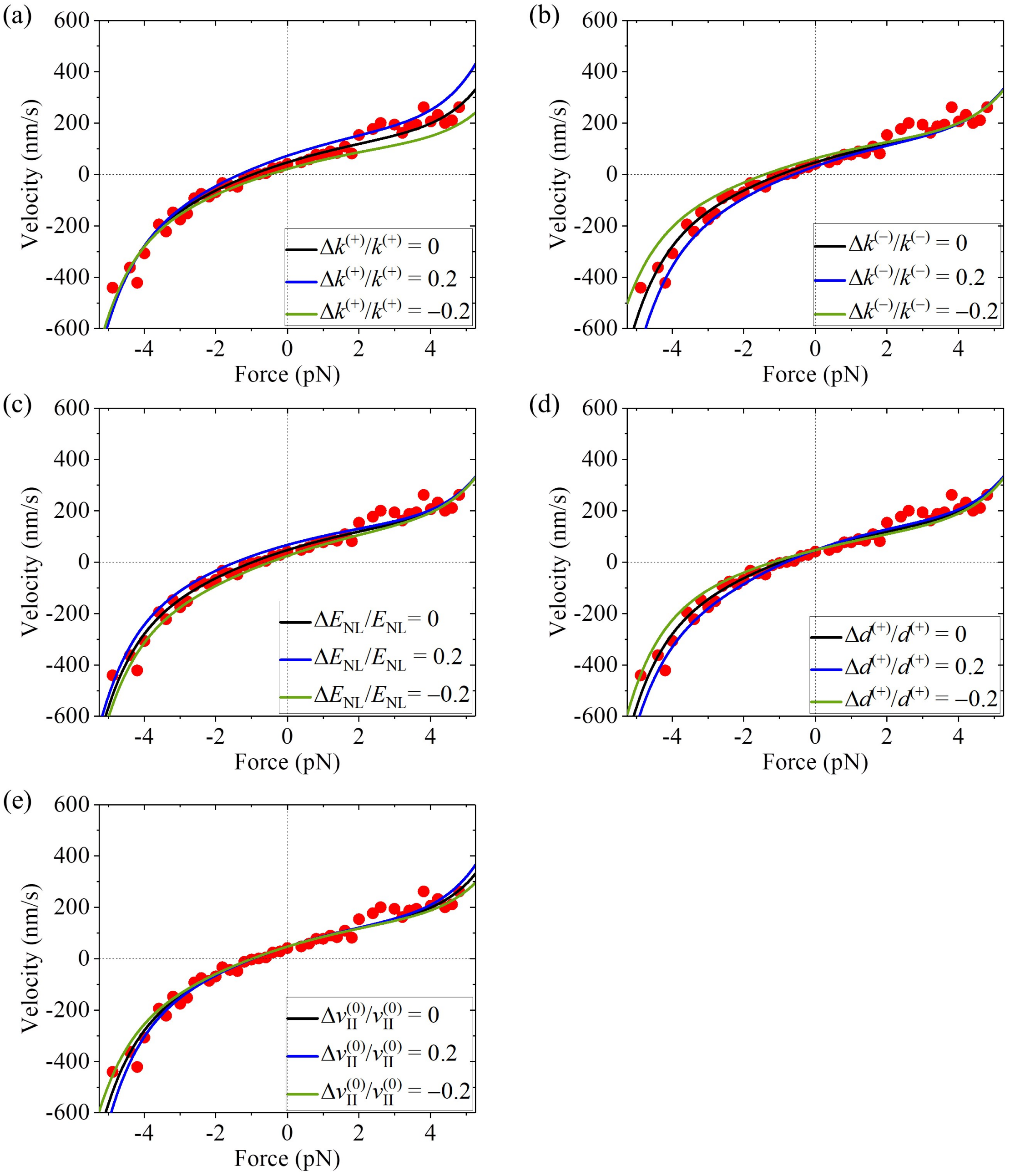

Figure S5. The sensitivity of theoretical results for the load dependence of velocity with including the slip to the variation of the adjustable parameter at saturating ATP. Lines are theoretical results calculated with parameter values listed in Table 1 except that one parameter value is changed. The symbols are experimental data taken from Jannasch et al. [S4]. **(a)** Effect of variation of individual $k^{(+)}$ by 20% on the results. **(b)** Effect of variation of individual $k^{(-)}$ by 20% on the results. **(c)** Effect of variation of individual E_{NL} by 10% on the results. **(d)** Effect of variation of individual $d^{(+)}$ by 20% on the results. **(d)** Effect of variation of individual $v_{II}^{(0)}$ by 20% on the results.

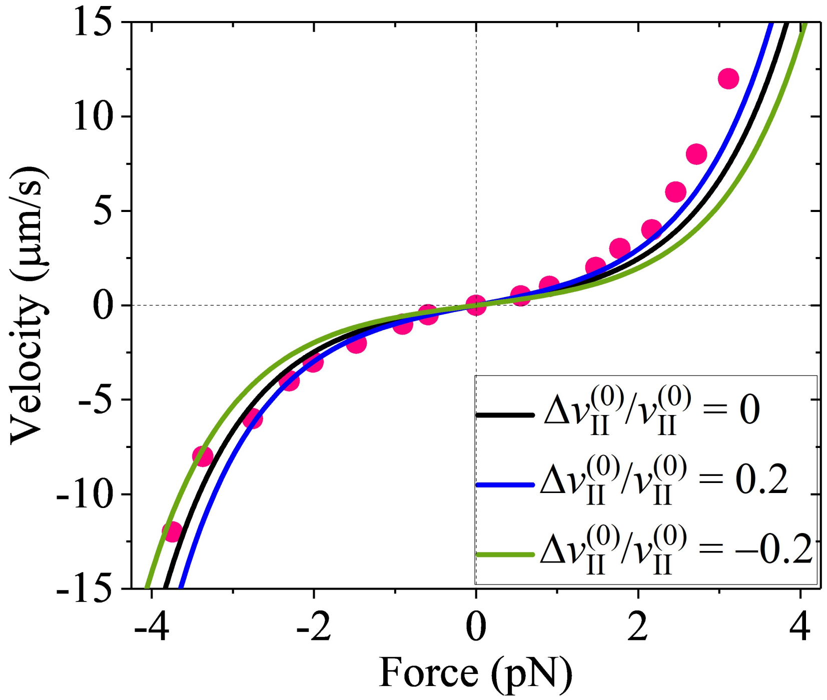

Figure S6. The sensitivity of theoretical results for the load dependence of slipping velocity to the variation of the adjustable parameter $v_{\rm II}^{(0)}$ by 20% at saturating ADP and no ATP. Lines are theoretical results calculated with parameter values listed in Table 1 except that the value of parameter $v_{\rm II}^{(0)}$ is changed. The symbols are experimental data taken from Bormuth et al. [S5].

References

[S1] Morikawa M., Yajima H., Nitta R., Inoue S., Ogura T., Sato C., Hirokawa N. (2015) X-ray and Cryo-EM structures reveal mutual conformational changes of Kinesin and GTP-state microtubules upon binding. EMBO J. 34, 1270−1286.

[S2] Shi, X.-X., Fu, Y.-B., Guo, S.-K., Wang, P.-Y., Chen, H., Xie, P. (2018) Investigating role of conformational changes of microtubule in regulating its binding affinity to kinesin by all-atom molecular dynamics simulation. Proteins 86, 1127–1139.

[S3] Xie P. (2020) Theoretical analysis of dynamics of kinesin molecular motors. ACS Omega 5, 5721−5730.

[S4] Jannasch A., Bormuth V., Storch M., Howard J., Schaffer E. (2013) Kinesin-8 is a low-force

motor protein with a weakly bound slip state. Biophys. J. 104, 2456–2464.
[S5] Bormuth V., Varga V., Howard J., Schaffer E. (2009) Protein friction limits diffusive and directed movements of kinesin motors on microtubules. Science 325, 870–873.
